# Supplementary material for: Purification Method of Extracellular Vesicles Derived from Human T-Cell Leukemia Virus Type 1-Infected Cells without Virions
Source: Viruses. 2024 Feb 4;16(2):249. doi: 10.3390/v16020249 (PMC10892183; doi:10.3390/v16020249)
Supplement: Supplementary file 1 [file viruses-16-00249-s001.zip › viruses-2751571-supplementary.pdf]

Supplementary Materials Table S1

A) Virus genomic RNA

```
Query Range: 647 - 1332
Sbjct Range: 1 - 686
686 bp, INT.Score: 2678, OPT.Score: 2678
Identity: 675 / 686 (98%)
Similarity: 675 / 686 (98%)
Gaps: 0 / 686 (0%)
Strand: Plus / Plus


Query  647  GTTCCACCCCTTTCCCTTTCATTCACGACTGACTGCCGGCTTGGCCCACGGCCAAGTACC  706
            ************************************************************
Sbjct    1  GTTCCACCCCTTTCCCTTTCATTCACGACTGACTGCCGGCTTGGCCCACGGCCAAGTACC  60


Query  707  GGCGACTCCGTTGGCTCGGAGCCAGCGACAGCCCATCCTATAGCACTCTCAGGAGAGAAA  766
            ************************************************************
Sbjct   61  GGCGACTCCGTTGGCTCGGAGCCAGCGACAGCCCATCCTATAGCACTCTCAGGAGAGAAA  120


Query  767  TTTAGTACACAGTTGGGGGCTCGTCCGGGATACGAGCGCCCCTTTATTCCCTAGGCAATG  826
            *********************************************************** 
Sbjct  121  TTTAGTACACAGTTGGGGGCTCGTCCGGGATACGAGCGCCCCTTTATTCCCTAGGCAATA  180


Query  827  GGCCAAATCTTTTCCCGTAGCGCTAGCCCTATTCCGCGACCGCCCCGGGGGCTGGCCGCT  886
            ************************************** *********************
Sbjct  181  GGCCAAATCTTTTCCCGTAGCGCTAGCCCTATTCCGCGGCCGCCCCGGGGGCTGGCCGCT  240


Query  887  CATCACTGGCTTAACTTCCTCCAGGCGGCATATCGCCTAGAACCCGGTCCCTCCAGTTAC  946
            *********************** ************************************
Sbjct  241  CATCACTGGCTTAACTTCCTCCAAGCGGCATATCGCCTAGAACCCGGTCCCTCCAGTTAC  300


Query  947  GATTTCCACCAGTTAAAAAAATTTCTTAAAATAGCTTTAGAAACACCGGCTCGGATCTGT  1006
            ************** **********************************   ********
Sbjct  301  GATTTCCACCAGTTGAAAAAATTTCTTAAAATAGCTTTAGAAACACCGGTCTGGATCTGT  360
```

```
Query  1007  CCCATTAACTACTCCCTCCTAGCCAGCCTACTCCCAAAAGGATACCCCGGCCGGGTGAAT  1066
             ************************************************************
Sbjct   361  CCCATTAACTACTCCCTCCTAGCCAGCCTACTCCCAAAAGGATACCCCGGCCGGGTGAAT  420

Query  1067  GAAATTTTACACATACTCATCCAAACCCAAGCCCAGATCCCGTCCCGTCCCGCGCCACCG  1126
             ************************************************************
Sbjct   421  GAAATTTTACACATACTCATCCAAACCCAAGCCCAGATCCCGTCCCGTCCCGCGCCACCG  480

Query  1127  CCGCCGTCATCCCCCACCCACGACCCCCCGGATTCTGATCCACAAATCCCCCCTCCCTAT  1186
             ************************************************************
Sbjct   481  CCGCCGTCATCCCCCACCCACGACCCCCCGGATTCTGATCCACAAATCCCCCCTCCCTAT  540

Query  1187  GTTGAGCCTACGGCCCCCCAAGTCCTTCCAGTCATGCATCCACATGGTGCTCCTCCTAAC  1246
             *********************** ************** *********** ***** ***
Sbjct   541  GTTGAGCCTACGGCCCCCCAAGTTCTTCCAGTCATGCACCCACATGGTGCCCCTCCCAAC  600

Query  1247  CATCGCCCATGGCAAATGAAAGACCTACAGGCCATTAAGCAAGAAGTCTCCCAAGCAGCC  1306
             ************************************************************
Sbjct   601  CATCGCCCATGGCAAATGAAAGACCTACAGGCCATTAAGCAAGAAGTCTCCCAAGCAGCC  660

Query  1307  CCTGGGAGCCCCCAGTTTATGCAGAC  1332
             **************************
Sbjct   661  CCTGGGAGCCCCCAGTTTATGCAGAC  686
```

Query Range: 6659 - 8345
Sbjct Range: 1 - 1686
1687 bp, INT.Score: 6282, OPT.Score: 6654
Identity: 1673 / 1687 (99%)
Similarity: 1673 / 1687 (99%)
Gaps: 1 / 1687 (0%)
Strand: Plus / Plus

```
Query  6659  CATCCCTGTAAACCAAGCACGCAATTATTGCAACCACATCGCCTCCAGCCTCCCCTGCCA  6718
             ************************************************************
Sbjct     1  CATCCCTGTAAACCAAGCACGCAATTATTGCAACCACATCGCCTCCAGCCTCCCCTGCCA  60

Query  6719  ATAATTAACCTCTCCCATCAAATCCTCCTTCTCCTGCAGCAACTTCCTCCGTTCAGCCTC  6778
             ************************************ ***********************
Sbjct    61  ATAATTAACCTCTCCCATCAAATCCTCCTTCTCCTG-AGCAACTTCCTCCGTTCAGCCTC  119

Query  6779  CAAGGACTCCACCTCGCCTTCCAACTGTCTAGTATAGCCATCAATCCCCAACTCCTGCAT  6838
             ************************************************************
Sbjct   120  CAAGGACTCCACCTCGCCTTCCAACTGTCTAGTATAGCCATCAATCCCCAACTCCTGCAT  179

Query  6839  TTTTTCTTTCCTAGCACTATGCTGTTTCGCCTTCTCAGCCCCTTGTCTCCACTTGCGCTC  6898
             ************************************************************
Sbjct   180  TTTTTCTTTCCTAGCACTATGCTGTTTCGCCTTCTCAGCCCCTTGTCTCCACTTGCGCTC  239

Query  6899  ACGGCGCTCCTGCTCTTCCTGCTTCCTCCTAGCGACGTCAGCGGCCTTCTTCTCCGCCCG  6958
             ************************ ****  ** **************************
Sbjct   240  ACGGCGCTCCTGCTCTTCCTGCTTTCTCCGGGCAACGTCAGCGGCCTTCTTCTCCGCCCG  299

Query  6959  CCTCCTGCGCCGTGCCTTCTCCTCTTCCTTCCTTTTCAAATACTCAGCGGTCTGCTTTTC  7018
             ************************************************  **********
Sbjct   300  CCTCCTGCGCCGTGCCTTCTCCTCTTCCTTCCTTTTCAAATACTCAGCAATCTGCTTTTC  359
```

```
Query  7019  CTCCTCTTTCTCCCGCTCTTTTTTTCGCTTCCTCTTCTCCTCAGCCCGTCGCTGCCGATC  7078
             ************************************************************
Sbjct   360  CTCCTCTTTCTCCCGCTCTTTTTTTCGCTTCCTCTTCTCCTCAGCCCGTCGCTGCCGATC  419

Query  7079  ACGATGCGTTTCCCCGCGAGGTGGCGCTTTCTCCCCTGGAGGGCCCCGTCGCAGCCGGCC  7138
             ****************************** *****************************
Sbjct   420  ACGATGCGTTTCCCCGCGAGGTGGCGCTTTTTCCCCTGGAGGGCCCCGTCGCAGCCGGCC  479

Query  7139  GCGGCTTTCCTCTTCTAAGGATAGCAAACCGTCAAGCACAGCTTCCTCCTCCTCCTTGTC  7198
             ************************************************************
Sbjct   480  GCGGCTTTCCTCTTCTAAGGATAGCAAACCGTCAAGCACAGCTTCCTCCTCCTCCTTGTC  539

Query  7199  CTTTAACTCTTCCTCCAAGGATAATAGCCCGTCCACCAATTCCTCCACCAGCAGGTCCTC  7258
             ************************************************************
Sbjct   540  CTTTAACTCTTCCTCCAAGGATAATAGCCCGTCCACCAATTCCTCCACCAGCAGGTCCTC  599

Query  7259  CGGGCATGACACAGGCAAGCATCGAAACAGCCCTGCAGATACAAAGTTAACCATGCTTAT  7318
             ******** ************************* *************************
Sbjct   600  CGGGCATGGCACAGGCAAGCATCGAAACAGCCCTACAGATACAAAGTTAACCATGCTTAT  659

Query  7319  TATCAGCCCACTTCCCAGGGTTTGGACAGAGTCTTCTTTTCGGATACCCAGTCTACGTGT  7378
             ************************************************************
Sbjct   660  TATCAGCCCACTTCCCAGGGTTTGGACAGAGTCTTCTTTTCGGATACCCAGTCTACGTGT  719
```

```
Query  7379  TTGGAGACTGTGTACAAGGCGACTGGTGCCCCATCTCTGGGGGACTATGTTCGGCCCGCC  7438
             ************************************************************
Sbjct   720  TTGGAGACTGTGTACAAGGCGACTGGTGCCCCATCTCTGGGGGACTATGTTCGGCCCGCC  779

Query  7439  TACATCGTCACGCCCTACTGGCCACCTGTCCAGAGCATCAGATCACCTGGGACCCCATCG  7498
             ************************************************************
Sbjct   780  TACATCGTCACGCCCTACTGGCCACCTGTCCAGAGCATCAGATCACCTGGGACCCCATCG  839

Query  7499  ATGGACGCGTTATCGGCTCAGCTCTACAGTTCCTTATCCCTCGACTCCCCTCCTTCCCCA  7558
             ************************************************************
Sbjct   840  ATGGACGCGTTATCGGCTCAGCTCTACAGTTCCTTATCCCTCGACTCCCCTCCTTCCCCA  899

Query  7559  CCCAGAGAACCTCTAAGACCCTCAAGGTCCTTACCCCGCCAATCACTCATACAACCCCCA  7618
             ************************************************************
Sbjct   900  CCCAGAGAACCTCTAAGACCCTCAAGGTCCTTACCCCGCCAATCACTCATACAACCCCCA  959

Query  7619  ACATTCCACCCTCCTTCCTCCAGGCCATGCGCAAATACTCCCCCTTCCGAAATGGATACA  7678
             ************************************************************
Sbjct   960  ACATTCCACCCTCCTTCCTCCAGGCCATGCGCAAATACTCCCCCTTCCGAAATGGATACA  1019

Query  7679  TGGAACCCACCCTTGGGCAGCACCTCCCAACCCTGTCTTTTCCAGACCCCGGACTCCGGC  7738
             ************************************************************
Sbjct  1020  TGGAACCCACCCTTGGGCAGCACCTCCCAACCCTGTCTTTTCCAGACCCCGGACTCCGGC  1079

Query  7739  CCCAAAACCTGTACACCCTCTGGGGAGGCTCCGTTGTCTGCATGTACCTCTACCAGCTTT  7798
             ************************************************************
Sbjct  1080  CCCAAAACCTGTACACCCTCTGGGGAGGCTCCGTTGTCTGCATGTACCTCTACCAGCTTT  1139

Query  7799  CCCCCCCCATCACCTGGCCCCTCCTGCCCCACGTGATTTTTTGCCACCCCGGCCAGCTCG  7858
             ************************************************************
Sbjct  1140  CCCCCCCCATCACCTGGCCCCTCCTGCCCCACGTGATTTTTTGCCACCCCGGCCAGCTCG  1199

Query  7859  GGGCCTTCCTCACCAATGTTCCCTACAAGCGAATAGAAGAACTCCTCTATAAAATTTCCC  7918
             ********************** *************************************
Sbjct  1200  GGGCCTTCCTCACCAATGTTCCGTACAAGCGAATAGAAGAACTCCTCTATAAAATTTCCC  1259

Query  7919  TCACCACAGGGGCCCTAATAATTCTACCCGAAGACTGTTTGCCCACCACCCTTTTCCAGC  7978
             * **********************************************************
Sbjct  1260  TTACCACAGGGGCCCTAATAATTCTACCCGAAGACTGTTTGCCCACCACCCTTTTCCAGC  1319
```

```
Query  7979  CTGCTAGGGCACCCGTCACGCTAACAGCCTGGCAAAACGGCCTCCTTCCGTTCCACTCAA  8038
             *** ********************************************************
Sbjct  1320  CTGTTAGGGCACCCGTCACGCTAACAGCCTGGCAAAACGGCCTCCTTCCGTTCCACTCAA  1379

Query  8039  CCCTCACCACTCCAGGCCTTATTTGGACATTTACCGATGGCACGCCTATGATTTCCGGGC  8098
             ************************************************************
Sbjct  1380  CCCTCACCACTCCAGGCCTTATTTGGACATTTACCGATGGCACGCCTATGATTTCCGGGC  1439

Query  8099  CCTGCCCTAAAGATGGCCAGCCATCTTTAGTACTACAGTCCTCCTCCTTTATATTTCACA  8158
             ************************************************************
Sbjct  1440  CCTGCCCTAAAGATGGCCAGCCATCTTTAGTACTACAGTCCTCCTCCTTTATATTTCACA  1499

Query  8159  AATTTCAAACCAAGGCCTACCACCCCTCATTTCTACTCTCACACGGCCTCATACAGTACT  8218
             ************************************************************
Sbjct  1500  AATTTCAAACCAAGGCCTACCACCCCTCATTTCTACTCTCACACGGCCTCATACAGTACT  1559

Query  8219  CTTCCTTTCATAGTTTACATCTCCTGTTTGAAGAATACACCAACATCCCCATTTCTCTAC  8278
             ************ ***********************************************
Sbjct  1560  CTTCCTTTCATAATTTACATCTCCTGTTTGAAGAATACACCAACATCCCCATTTCTCTAC  1619

Query  8279  TTTTTAACGAAAAAGAGGCAGATGACAATGACCATGAGCCCCAAATATCCCCCGGGGGCT  8338
             ************************************************************
Sbjct  1620  TTTTTAACGAAAAAGAGGCAGATGACAATGACCATGAGCCCCAAATATCCCCCGGGGGCT  1679

Query  8339  TAGAGCC  8345
             *******
Sbjct  1680  TAGAGCC  1686
```

B) Defective virus genomic RNA

```
Query Range: 1318 - 1332
Sbjct Range: 1 - 15
15 bp, INT.Score: 60, OPT.Score: 60
Identity: 15 / 15 (100%)
Similarity: 15 / 15 (100%)
Gaps: 0 / 15 (0%)
Strand: Plus / Plus


Query  1318  CCAGTTTATGCAGAC  1332
             ***************
Sbjct     1  CCAGTTTATGCAGAC  15
```

```
Query Range: 6659 - 8345
Sbjct Range: 1 - 1687
1687 bp, INT.Score: 6688, OPT.Score: 6688
Identity: 1677 / 1687 (99%)
Similarity: 1677 / 1687 (99%)
Gaps: 0 / 1687 (0%)
Strand: Plus / Plus

Query  6659  CATCCCTGTAAACCAAGCACGCAATTATTGCAACCACATCGCCTCCAGCCTCCCCTGCCA  6718
             ********************  **************************************
Sbjct     1  CATCCCTGTAAACCAAGCACATAATTATTGCAACCACATCGCCTCCAGCCTCCCCTGCCA  60

Query  6719  ATAATTAACCTCTCCCATCAAATCCTCCTTCTCCTGCAGCAACTTCCTCCGTTCAGCCTC  6778
             ****************** *****************************************
Sbjct    61  ATAATTAACCTCTCCCATTAAATCCTCCTTCTCCTGCAGCAACTTCCTCCGTTCAGCCTC  120

Query  6779  CAAGGACTCCACCTCGCCTTCCAACTGTCTAGTATAGCCATCAATCCCCAACTCCTGCAT  6838
             ************************************************************
Sbjct   121  CAAGGACTCCACCTCGCCTTCCAACTGTCTAGTATAGCCATCAATCCCCAACTCCTGCAT  180

Query  6839  TTTTTCTTTCCTAGCACTATGCTGTTTCGCCTTCTCAGCCCCTTGTCTCCACTTGCGCTC  6898
             ************************************************************
Sbjct   181  TTTTTCTTTCCTAGCACTATGCTGTTTCGCCTTCTCAGCCCCTTGTCTCCACTTGCGCTC  240

Query  6899  ACGGCGCTCCTGCTCTTCCTGCTTCCTCCTAGCGACGTCAGCGGCCTTCTTCTCCGCCCG  6958
             ************************ ***********************************
Sbjct   241  ACGGCGCTCCTGCTCTTCCTGCTTTCTCCTAGCGACGTCAGCGGCCTTCTTCTCCGCCCG  300

Query  6959  CCTCCTGCGCCGTGCCTTCTCCTCTTCCTTCCTTTTCAAATACTCAGCGGTCTGCTTTTC  7018
             ************************************************************
Sbjct   301  CCTCCTGCGCCGTGCCTTCTCCTCTTCCTTCCTTTTCAAATACTCAGCGGTCTGCTTTTC  360
```

```
Query  7019  CTCCTCTTTCTCCCGCTCTTTTTTTCGCTTCCTCTTCTCCTCAGCCCGTCGCTGCCGATC  7078
             ************************************************************
Sbjct   361  CTCCTCTTTCTCCCGCTCTTTTTTTCGCTTCCTCTTCTCCTCAGCCCGTCGCTGCCGATC  420

Query  7079  ACGATGCGTTTCCCCGCGAGGTGGCGCTTTCTCCCCTGGAGGGCCCCGTCGCAGCCGGCC  7138
             ****************************** *****************************
Sbjct   421  ACGATGCGTTTCCCCGCGAGGTGGCGCTTTTTCCCCTGGAGGGCCCCGTCGCAGCCGGCC  480

Query  7139  GCGGCTTTCCTCTTCTAAGGATAGCAAACCGTCAAGCACAGCTTCCTCCTCCTCCTTGTC  7198
             ************************************************************
Sbjct   481  GCGGCTTTCCTCTTCTAAGGATAGCAAACCGTCAAGCACAGCTTCCTCCTCCTCCTTGTC  540

Query  7199  CTTTAACTCTTCCTCCAAGGATAATAGCCCGTCCACCAATTCCTCCACCAGCAGGTCCTC  7258
             ************************************************************
Sbjct   541  CTTTAACTCTTCCTCCAAGGATAATAGCCCGTCCACCAATTCCTCCACCAGCAGGTCCTC  600

Query  7259  CGGGCATGACACAGGCAAGCATCGAAACAGCCCTGCAGATACAAAGTTAACCATGCTTAT  7318
             ******** ************************* *************************
Sbjct   601  CGGGCATGGCACAGGCAAGCATCGAAACAGCCCTACAGATACAAAGTTAACCATGCTTAT  660

Query  7319  TATCAGCCCACTTCCCAGGGTTTGGACAGAGTCTTCTTTTCGGATACCCAGTCTACGTGT  7378
             ************************************************************
Sbjct   661  TATCAGCCCACTTCCCAGGGTTTGGACAGAGTCTTCTTTTCGGATACCCAGTCTACGTGT  720

Query  7379  TTGGAGACTGTGTACAAGGCGACTGGTGCCCCATCTCTGGGGGACTATGTTCGGCCCGCC  7438
             ************************************************************
Sbjct   721  TTGGAGACTGTGTACAAGGCGACTGGTGCCCCATCTCTGGGGGACTATGTTCGGCCCGCC  780

Query  7439  TACATCGTCACGCCCTACTGGCCACCTGTCCAGAGCATCAGATCACCTGGGACCCCATCG  7498
             ************************************************************
Sbjct   781  TACATCGTCACGCCCTACTGGCCACCTGTCCAGAGCATCAGATCACCTGGGACCCCATCG  840

Query  7499  ATGGACGCGTTATCGGCTCAGCTCTACAGTTCCTTATCCCTCGACTCCCCTCCTTCCCCA  7558
             ************************************************************
Sbjct   841  ATGGACGCGTTATCGGCTCAGCTCTACAGTTCCTTATCCCTCGACTCCCCTCCTTCCCCA  900

Query  7559  CCCAGAGAACCTCTAAGACCCTCAAGGTCCTTACCCCGCCAATCACTCATACAACCCCCA  7618
             ************************************************************
Sbjct   901  CCCAGAGAACCTCTAAGACCCTCAAGGTCCTTACCCCGCCAATCACTCATACAACCCCCA  960
```

```
Query  7619  ACATTCCACCCTCCTTCCTCCAGGCCATGCGCAAATACTCCCCCTTCCGAAATGGATACA  7678
             ************************************************************
Sbjct   961  ACATTCCACCCTCCTTCCTCCAGGCCATGCGCAAATACTCCCCCTTCCGAAATGGATACA  1020

Query  7679  TGGAACCCACCCTTGGGCAGCACCTCCCAACCCTGTCTTTTCCAGACCCCGGACTCCGGC  7738
             ************************************************************
Sbjct  1021  TGGAACCCACCCTTGGGCAGCACCTCCCAACCCTGTCTTTTCCAGACCCCGGACTCCGGC  1080

Query  7739  CCCAAAACCTGTACACCCTCTGGGGAGGCTCCGTTGTCTGCATGTACCTCTACCAGCTTT  7798
             ************************************************************
Sbjct  1081  CCCAAAACCTGTACACCCTCTGGGGAGGCTCCGTTGTCTGCATGTACCTCTACCAGCTTT  1140

Query  7799  CCCCCCCCATCACCTGGCCCCTCCTGCCCCACGTGATTTTTTGCCACCCCGGCCAGCTCG  7858
             ************************************************************
Sbjct  1141  CCCCCCCCATCACCTGGCCCCTCCTGCCCCACGTGATTTTTTGCCACCCCGGCCAGCTCG  1200

Query  7859  GGGCCTTCCTCACCAATGTTCCCTACAAGCGAATAGAAGAACTCCTCTATAAAATTTCCC  7918
             ********************** *************************************
Sbjct  1201  GGGCCTTCCTCACCAATGTTCCGTACAAGCGAATAGAAGAACTCCTCTATAAAATTTCCC  1260

Query  7919  TCACCACAGGGGCCCTAATAATTCTACCCGAAGACTGTTTGCCCACCACCCTTTTCCAGC  7978
             * **********************************************************
Sbjct  1261  TTACCACAGGGGCCCTAATAATTCTACCCGAAGACTGTTTGCCCACCACCCTTTTCCAGC  1320

Query  7979  CTGCTAGGGCACCCGTCACGCTAACAGCCTGGCAAAACGGCCTCCTTCCGTTCCACTCAA  8038
             ************************************************************
Sbjct  1321  CTGCTAGGGCACCCGTCACGCTAACAGCCTGGCAAAACGGCCTCCTTCCGTTCCACTCAA  1380

Query  8039  CCCTCACCACTCCAGGCCTTATTTGGACATTTACCGATGGCACGCCTATGATTTCCGGGC  8098
             ************************************************************
Sbjct  1381  CCCTCACCACTCCAGGCCTTATTTGGACATTTACCGATGGCACGCCTATGATTTCCGGGC  1440

Query  8099  CCTGCCCTAAAGATGGCCAGCCATCTTTAGTACTACAGTCCTCCTCCTTTATATTTCACA  8158
             ************************************************************
Sbjct  1441  CCTGCCCTAAAGATGGCCAGCCATCTTTAGTACTACAGTCCTCCTCCTTTATATTTCACA  1500

Query  8159  AATTTCAAACCAAGGCCTACCACCCCTCATTTCTACTCTCACACGGCCTCATACAGTACT  8218
             ************************************************************
Sbjct  1501  AATTTCAAACCAAGGCCTACCACCCCTCATTTCTACTCTCACACGGCCTCATACAGTACT  1560
```

```
Query  8219  CTTCCTTTCATAGTTTACATCTCCTGTTTGAAGAATACACCAACATCCCCATTTCTCTAC  8278
             ************ ***********************************************
Sbjct  1561  CTTCCTTTCATAATTTACATCTCCTGTTTGAAGAATACACCAACATCCCCATTTCTCTAC  1620

Query  8279  TTTTTAACGAAAAAGAGGCAGATGACAATGACCATGAGCCCCAAATATCCCCCGGGGGCT  8338
             ************************************************************
Sbjct  1621  TTTTTAACGAAAAAGAGGCAGATGACAATGACCATGAGCCCCAAATATCCCCCGGGGGCT  1680

Query  8339  TAGAGCC  8345
             *******
Sbjct  1681  TAGAGCC  1687
```

C) Complete virus genomic RNA

```
Query Range: 647 - 3085
Sbjct Range: 1 - 2439
2439 bp, INT.Score: 9645, OPT.Score: 9645
Identity: 2420 / 2439 (99%)
Similarity: 2421 / 2439 (99%)
Gaps: 0 / 2439 (0%)
Strand: Plus / Plus


Query   647  GTTCCACCCCTTTCCCTTTCATTCACGACTGACTGCCGGCTTGGCCCACGGCCAAGTACC  706
             ************************************************************
Sbjct     1  GTTCCACCCCTTTCCCTTTCATTCACGACTGACTGCCGGCTTGGCCCACGGCCAAGTACC  60


Query   707  GGCGACTCCGTTGGCTCGGAGCCAGCGACAGCCCATCCTATAGCACTCTCAGGAGAGAAA  766
             ************************************************************
Sbjct    61  GGCGACTCCGTTGGCTCGGAGCCAGCGACAGCCCATCCTATAGCACTCTCAGGAGAGAAA  120


Query   767  TTTAGTACACAGTTGGGGGCTCGTCCGGGATACGAGCGCCCCTTTATTCCCTAGGCAATG  826
             ************************************************************
Sbjct   121  TTTAGTACACAGTTGGGGGCTCGTCCGGGATACGAGCGCCCCTTTATTCCCTAGGCAATG  180


Query   827  GGCCAAATCTTTTCCCGTAGCGCTAGCCCTATTCCGCGACCGCCCCGGGGGCTGGCCGCT  886
             ************************************** *********************
Sbjct   181  GGCCAAATCTTTTCCCGTAGCGCTAGCCCTATTCCGCGGCCGCCCCGGGGGCTGGCCGCT  240


Query   887  CATCACTGGCTTAACTTCCTCCAGGCGGCATATCGCCTAGAACCCGGTCCCTCCAGTTAC  946
             *********************** ************************************
Sbjct   241  CATCACTGGCTTAACTTCCTCCAAGCGGCATATCGCCTAGAACCCGGTCCCTCCAGTTAC  300


Query   947  GATTTCCACCAGTTAAAAAAATTTCTTAAAATAGCTTTAGAAACACCGGCTCGGATCTGT  1006
             ******************************************* ****************
Sbjct   301  GATTTCCACCAGTTAAAAAAATTTCTTAAAATAGCTTTAGAAAGACCGGCTCGGATCTGT  360
```

```
Query  1007  CCCATTAACTACTCCCTCCTAGCCAGCCTACTCCCAAAAGGATACCCCGGCCGGGTGAAT  1066
             ******* ****************************************************
Sbjct   361  CCCATTAGCTACTCCCTCCTAGCCAGCCTACTCCCAAAAGGATACCCCGGCCGGGTGAAT  420

Query  1067  GAAATTTTACACATACTCATCCAAACCCAAGCCCAGATCCCGTCCCGTCCCGCGCCACCG  1126
             ************************************************************
Sbjct   421  GAAATTTTACACATACTCATCCAAACCCAAGCCCAGATCCCGTCCCGTCCCGCGCCACCG  480

Query  1127  CCGCCGTCATCCCCCACCCACGACCCCCCGGATTCTGATCCACAAATCCCCCCTCCCTAT  1186
             ************************************************************
Sbjct   481  CCGCCGTCATCCCCCACCCACGACCCCCCGGATTCTGATCCACAAATCCCCCCTCCCTAT  540

Query  1187  GTTGAGCCTACGGCCCCCCAAGTCCTTCCAGTCATGCATCCACATGGTGCTCCTCCTAAC  1246
             *********************** ********* **** *********************
Sbjct   541  GTTGAGCCTACGGCCCCCCAAGTTCTTCCAGTCGTGCACCCACATGGTGCTCCTCCTAAC  600

Query  1247  CATCGCCCATGGCAAATGAAAGACCTACAGGCCATTAAGCAAGAAGTCTCCCAAGCAGCC  1306
             *******************************.****************************
Sbjct   601  CATCGCCCATGGCAAATGAAAGACCTACAGGNCATTAAGCAAGAAGTCTCCCAAGCAGCC  660

Query  1307  CCTGGGAGCCCCCAGTTTATGCAGACCATCCGGCTTGCGGTGCAGCAGTTTGACCCCACT  1366
             ************************************************************
Sbjct   661  CCTGGGAGCCCCCAGTTTATGCAGACCATCCGGCTTGCGGTGCAGCAGTTTGACCCCACT  720
```

```
Query  1367  GCCAAAGACCTCCAAGACCTCCTGCAGTACCTTTGCTCCTCCCTCGTGGCTTCCCTCCAT  1426
             ************************************************************
Sbjct   721  GCCAAAGACCTCCAAGACCTCCTGCAGTACCTTTGCTCCTCCCTCGTGGCTTCCCTCCAT  780

Query  1427  CACCAGCAGCTAGATAGCCTTATATCAGAGGCCGAAACCCGAGGTATTACAGGTTATAAC  1486
             ************************************************************
Sbjct   781  CACCAGCAGCTAGATAGCCTTATATCAGAGGCCGAAACCCGAGGTATTACAGGTTATAAC  840

Query  1487  CCATTAGCCGGTCCCCTCCGTGTCCAAGCCAACAATCCACAACAACAAGGATTAAGGCGA  1546
             ************************************************************
Sbjct   841  CCATTAGCCGGTCCCCTCCGTGTCCAAGCCAACAATCCACAACAACAAGGATTAAGGCGA  900

Query  1547  GAATACCAGCAACTCTGGCTCGCCGCCTTCGCCGCCCTGCCGGGGAGTGCCAAAGACCCT  1606
             ***************************************** ******************
Sbjct   901  GAATACCAGCAACTCTGGCTCGCCGCCTTCGCCGCCCTGCCAGGGAGTGCCAAAGACCCT  960

Query  1607  TCCTGGGCCTCTATCCTCCAAGGCCTGGAGGAGCCTTACCACGCCTTCGTAGAACGCCTC  1666
             ****************************** *****************************
Sbjct   961  TCCTGGGCCTCTATCCTCCAAGGCCTGGAGCAGCCTTACCACGCCTTCGTAGAACGCCTC  1020

Query  1667  AACATAGCTCTTGACAATGGGCTGCCAGAAGGCACGCCCAAAGACCCCATCTTACGTTCC  1726
             ************************************************** *********
Sbjct  1021  AACATAGCTCTTGACAATGGGCTGCCAGAAGGCACGCCCAAAGACCCCATTTTACGTTCC  1080

Query  1727  TTAGCCTACTCCAATGCAAACAAAGAATGCCAAAAATTACTACAGGCCCGAGGACACACT  1786
             ******* ****************************************************
Sbjct  1081  TTAGCCTTCTCCAATGCAAACAAAGAATGCCAAAAATTACTACAGGCCCGAGGACACACT  1140

Query  1787  AATAGCCCTCTAGGAGATATGTTGCGGGCTTGTCAGACCTGGACCCCCAAAGACAAAACC  1846
             ************************************************************
Sbjct  1141  AATAGCCCTCTAGGAGATATGTTGCGGGCTTGTCAGACCTGGACCCCCAAAGACAAAACC  1200

Query  1847  AAAGTGTTAGTTGTCCAGCCTAAAAAACCCCCCCCAAATCAGCCGTGCTTCCGGTGCGGG  1906
             ************************************************************
Sbjct  1201  AAAGTGTTAGTTGTCCAGCCTAAAAAACCCCCCCCAAATCAGCCGTGCTTCCGGTGCGGG  1260

Query  1907  AAAGCAGGCCACTGGAGTCGGGACTGCACTCAGCCTCGTCCCCCCCCCGGGCCATGCCCC  1966
             *********************************** ************************
Sbjct  1261  AAAGCAGGCCACTGGAGTCGGGACTGCACTCAGCCCCGTCCCCCCCCCGGGCCATGCCCC  1320
```

```
Query  1967  CTATGTCAAGACCCAACTCACTGGAAGCGAGACTGCCCCCGCCTAAAGCCCACTATCCCA  2026
             ************************************************************
Sbjct  1321  CTATGTCAAGACCCAACTCACTGGAAGCGAGACTGCCCCCGCCTAAAGCCCACTATCCCA  1380

Query  2027  GAACCAGAGCCAGAGGAAGATGCCCTCCTATTAGACCTCCCCGCTGACATCCCACACCCA  2086
             ************************************************************
Sbjct  1381  GAACCAGAGCCAGAGGAAGATGCCCTCCTATTAGACCTCCCCGCTGACATCCCACACCCA  1440

Query  2087  AAAAACTTCATAGGGGGGGAGGTTTAACCTCCCCCCCCACATTACAGCAAGTCCTTCCTA  2146
             ************************************************************
Sbjct  1441  AAAAACTTCATAGGGGGGGAGGTTTAACCTCCCCCCCCACATTACAGCAAGTCCTTCCTA  1500

Query  2147  ACCAAGACCCAGCATCTATTCTGCCAGTTATACCGTTAGATCCCGCCCGTCGGCCCGTAA  2206
             ************************************************************
Sbjct  1501  ACCAAGACCCAGCATCTATTCTGCCAGTTATACCGTTAGATCCCGCCCGTCGGCCCGTAA  1560

Query  2207  TTAAAGCCCAGGTTGACACCCAGACCAGCCACCCAAAGACTATCGAAGCTTTACTAGATA  2266
             ************************************************************
Sbjct  1561  TTAAAGCCCAGGTTGACACCCAGACCAGCCACCCAAAGACTATCGAAGCTTTACTAGATA  1620

Query  2267  CAGGAGCAGACATGACAGTCCTTCCGATAGCCTTGTTCTCAAGTAATACTCCCCTCAAAA  2326
             ************************************************************
Sbjct  1621  CAGGAGCAGACATGACAGTCCTTCCGATAGCCTTGTTCTCAAGTAATACTCCCCTCAAAA  1680

Query  2327  ATACATCCGTATTAGGGGCAGGGGGCCAAACCCAAGATCACTTTAAGCTCACCTCCCTTC  2386
             ************************************************************
Sbjct  1681  ATACATCCGTATTAGGGGCAGGGGGCCAAACCCAAGATCACTTTAAGCTCACCTCCCTTC  1740

Query  2387  CTGTGCTAATACGCCTCCCTTTCCGGACAACGCCTATTGTTTTAACATCTTGCCTAGTTG  2446
             ************************************************************
Sbjct  1741  CTGTGCTAATACGCCTCCCTTTCCGGACAACGCCTATTGTTTTAACATCTTGCCTAGTTG  1800

Query  2447  ATACCAAAAACAACTAGGCCATCATAGGTCGTGATGCCTTACAACAATGCCAAGGCGTCC  2506
             *************** ********************************************
Sbjct  1801  ATACCAAAAACAACTGGGCCATCATAGGTCGTGATGCCTTACAACAATGCCAAGGCGTCC  1860

Query  2507  TGTACCTCCCTGAGGCAAAAAGGCCGCCTGTAATCTTGCCAATACAGGCGCCAGCCGTCC  2566
             ************************************************************
Sbjct  1861  TGTACCTCCCTGAGGCAAAAAGGCCGCCTGTAATCTTGCCAATACAGGCGCCAGCCGTCC  1920
```

```
Query  2567  TTGGGCTAGAACACCTCCCAAGGCCCCCCGAAATCAGCCAGTTCCCTTTAAACCAGAACG  2626
             ************************************************************
Sbjct  1921  TTGGGCTAGAACACCTCCCAAGGCCCCCCGAAATCAGCCAGTTCCCTTTAAACCAGAACG  1980

Query  2627  CCTCCAGGCCTTGCAACACTTGGTCCGGAAGGCCCTGGAGGCAGGCCATATCGAACCCTA  2686
             ******************************************************* ****
Sbjct  1981  CCTCCAGGCCTTGCAACACTTGGTCCGGAAGGCCCTGGAGGCAGGCCATATCGAATCCTA  2040

Query  2687  CACCGGGCCAGGGAATAACCCAGTATTCCCAGTTAAAAAGGCCAATGGAACCTGGCGATT  2746
             ************ ***********************************************
Sbjct  2041  CACCGGGCCAGGAAATAACCCAGTATTCCCAGTTAAAAAGGCCAATGGAACCTGGCGATT  2100

Query  2747  CATCCACGACCTGCGGGCCACTAACTCTCTAACCATAGATCTCTCATCATCTTCCCCCGG  2806
             *************************************** ********************
Sbjct  2101  CATCCACGACCTGCGGGCCACTAACTCTCTAACCATAGACCTCTCATCATCTTCCCCCGG  2160

Query  2807  GCCCCCTGACTTGTCCAGCCTGCCAACCACACTAGCCCACTTGCAAACTATAGACCTTAG  2866
             *************************** ******************************** 
Sbjct  2161  GCCCCCTGACTTGTCCAGCCTGCCAACTACACTAGCCCACTTGCAAACTATAGACCTTAA  2220

Query  2867  AGACGCCTTTTTCCAAATCCCCTTACCTAAACAGTTCCAGCCCTACTTTGCTTTCACTGT  2926
             ************************************************************
Sbjct  2221  AGACGCCTTTTTCCAAATCCCCTTACCTAAACAGTTCCAGCCCTACTTTGCTTTCACTGT  2280

Query  2927  CCCACAGCAGTGTAACTACGGCCCCGGCACTAGATACGCCTGGAAAGTACTACCCCAAGG  2986
             ************************************************************
Sbjct  2281  CCCACAGCAGTGTAACTACGGCCCCGGCACTAGATACGCCTGGAAAGTACTACCCCAAGG  2340

Query  2987  GTTTAAAAATAGTCCCACCCTGTTCGAAATGCAGCTGGCCCATATCCTGCAGCCCATTCG  3046
             ************************************************************
Sbjct  2341  GTTTAAAAATAGTCCCACCCTGTTCGAAATGCAGCTGGCCCATATCCTGCAGCCCATTCG  2400

Query  3047  GCAAGCTTTCCCCCAATGCACTATTCTTCAGTACATGGA  3085
             ***************************************
Sbjct  2401  GCAAGCTTTCCCCCAATGCACTATTCTTCAGTACATGGA  2439
```
